# Unreported catches, impact of whaling and current status of blue whales in the South European Atlantic Shelf

**DOI:** 10.1038/s41598-022-09570-6

**Published:** 2022-03-31

**Authors:** Alex Aguilar, Asunción Borrell

**Affiliations:** 1 Department of Evolutionary Biology, Ecology and Environmental Sciences, Faculty of Biology, Universitat de Barcelona, Av. Diagonal, 643, 08028 Barcelona, Spain; 2 Institut de Recerca de la Biodiversitat (IRBio), Universitat de Barcelona, 08028 Barcelona, Spain

**Keywords:** Zoology, Environmental sciences

## Abstract

The North Atlantic blue whale was depleted by modern whaling and it is still considered to be highly endangered. Despite its protection in 1954, catches continued in the South European Atlantic Shelf (SEAS) region and immediately adjacent waters until 1979. We compiled catches and investigate abundance trends in the region using original data from whaling (1921–1985) and scientific surveys around the last years of exploitation (1981–1987). The struck and lost rate was estimated at 3.2% for sperm whales and 2.3% for baleen whales. The compiled records include 60 catches, with an additional 1–2 blue whales likely struck and lost. From these, 29 individuals had been correctly reported as blue whales but 31 were mislabelled as fin whales. After correcting for loss rates, the number of blue whales killed in the region was estimated at 61 in 55 years (1.12 individuals/year). The data from the 1950s shows some oversized fin whales but it is unclear whether they are due to an incorrect reporting of species or to incorrect measurements, so it cannot be discarded that the actual number of blue whales caught was slightly higher than estimated. Mean body length of reported blue whales was lower than in higher latitudes of the North Atlantic, probably reflecting geographical stratification with higher proportion of immatures in the SEAS. The ratio between catches or sightings of blue whales and those of fin whales was 5.9% in the southern part of the SEAS previous to exploitation, it declined to 0.02–0.18% in the 1920s, and increased thereafter up to 1.6% in the 1980–1990s. Taking as reference the population size of fin whales in the SEAS, that of blue whales at the end of the 1980s can be guessed to be at ca337-497 individuals. Considering accepted population estimates in other areas as well as the observed rates of increase, current abundance is thought to be over a thousand whales in the SEAs and at in the order of 4000–5000 individuals for the whole eastern North Atlantic basin.

## Introduction

Because of its massive body size and economic profitability, the blue whale (*Balaenoptera musculus*) was the most sought after species when modern whaling started its activities in the 1860s. The first catches were made in North Norway, but soon operations extended to other locations of the North Atlantic before they spread in the first years of the twentieth century to the North Pacific and the Antarctic Oceans^1^. However, in the North Atlantic the blue whale populations were relatively small and catches rapidly plummeted over the course of several decades of exploitation^2,3^. International Whaling Commission (IWC) catch statistics, built on the records earlier assembled by the Bureau of Whaling Statistics (BIWS) between 1930 and 1986, and completed and corrected thereafter^4^, only reflect 10,747 catches in the North Atlantic during 1864–2019, although actual numbers probably ranged between 15,000 and 20,000 because in the first years of exploitation some blue whales were caught and not reported^5^. Despite these numbers being low compared to those from other species (for example the statistics record the catch of 81,173 fin whales, *Balaenoptera physalus*, during the same period), already at the start of the twentieth century the species was residual in most areas of the North Atlantic Ocean and was substituted by the fin whale as a main target^6^. When the pioneer Convention for the Regulation of Whaling was signed in Geneva in 1931 there was already a clear perception that several blue whale stocks had been dramatically impacted and, in 1936, exploitation of the species was prohibited between the Equator and 40° S latitude, the fringe of waters where it was believed to reproduce^7^. In 1946 the International Whaling Commission (IWC) was established, and in its meeting in Tokyo in 1954 approved a ban on the exploitation of blue whales in the North Atlantic Ocean^8^. Iceland and Denmark initially objected the decision and were the only IWC-member countries that for some years continued carrying out its exploitation. However, blue whales were so scarce that their combined catch was only 5–13 whales per season^9^. In 1960 these two countries withdrew their objection and agreed to stop killing blue whales, and thus the complete protection of the species in the North Atlantic came into force amongst all IWC member nations^10^. The few catches of blue whales recorded thereafter were only scattered mistaken captures catalogued as infractions, which totalled 4 individuals during the period 1960–1966^11^.

**Figure 1:**
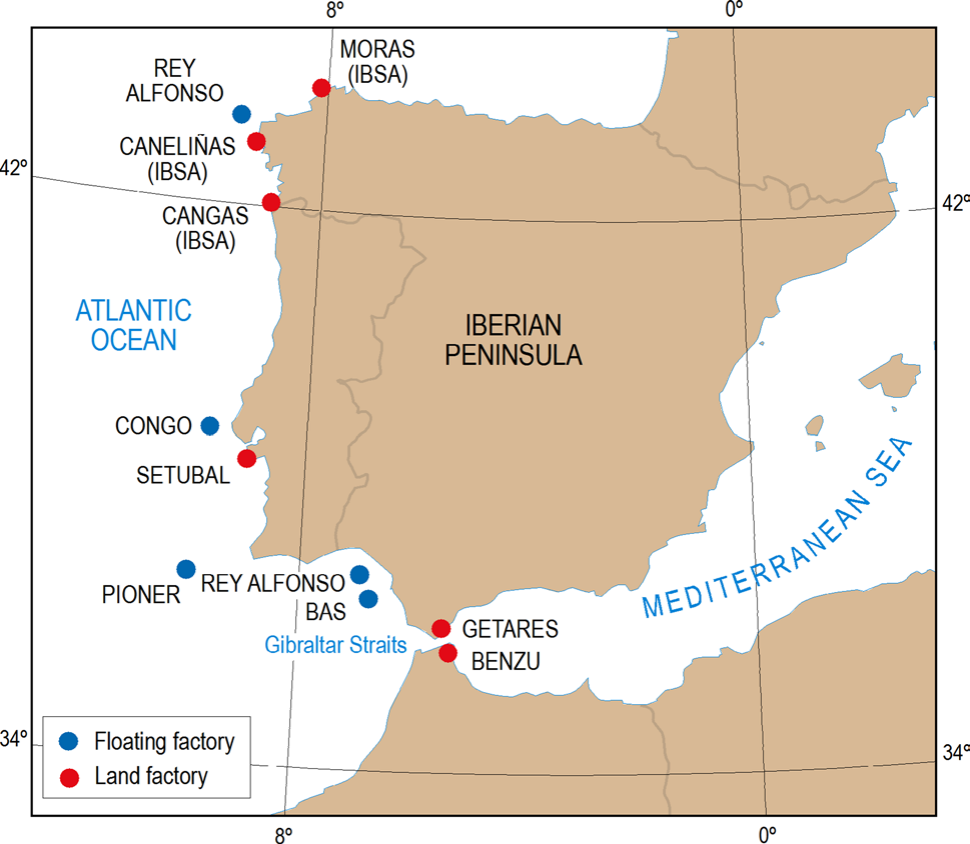
Location of the whaling stations that operated around the Iberian Peninsula. Figure generated by Albert Martinez with the program Freehand 10.

At the time that IWC passed its ban on the exploitation of blue whales, all North Atlantic whaling countries adhered to the International Convention for the Regulation of Whaling (ICRW) with the exception of Portugal and Spain. In both countries modern whaling had started in the 1920s and continued with some interruptions until the moratorium on commercial whaling adopted by the IWC came into force in 1986. Operations were conducted from several whaling factories: the Getares land station (1921–1926 and 1950–1959), the *Rey Alfonso* (1924–1926), *Bas* (1924), *Congo* (1925), and *Pioner* (1934) floating factories, the Benzú land station (1947–1954), the Setúbal land station (1925–1927 and 1944–1951), the *Industria Ballenera SA* (IBSA) land stations of Cangas, Caneliñas and Morás (1951–1985), the *Sierra* unregulated floating factory (1977–1979), as well a number of small land stations in the Azores (1921–1987) and Madeira (1941–1981)^12–14^ (see Fig. 1 for locations around the Iberian Peninsula). The blue whale ban of 1954 did not indeed affect Portuguese stations because the then remaining land factories in Madeira and the Azores mostly focused on sperm whales (*Physeter macrocephalus*) and their occasional catches of rorquals never included blue whales^11^. Spain, conversely, had at that time three operating land stations run by the IBSA company (Caneliñas, Cangas and Morás) that strongly relied on the exploitation of Balaenopteridae whales and, although besides the sperm whale the main exploited species was the fin whale, they also took sei whales (*Balaenoptera borealis*) and, to a lesser extent, blue and humpback whales (*Megaptera novaeangliae*)^14^. Not being a signatory to the Convention, Spain did not have to abide by the IWC regulations and thus ignored the prohibition on catching blue whales.

In order to prevent these and other whaling operations from escaping its jurisdiction, in 1978 the IWC agreed that member countries could not import products from non-member countries. Spain, which was then exporting most of its whaling products to Japan, saw its business in danger and was left with no choice but to adhere to the IWC. In 1979 it joined the organization and, from then on, it stopped capturing protected species, including blue whales^14^. As a result, the protection of the species became thereafter fully effective in the North Atlantic and no catches have been recorded since that time.

In the North Atlantic, blue whales are distributed from the edge of the pack ice to tropical and subtropical waters^15^. The variation in abundance trends in different areas that had been subject to exploitation, with asynchronous local depletions caused by the excessive takes, suggests that the North Atlantic Ocean metapopulation is structured into different units with at least two discrete subpopulations, one occupying the western basin and another occupying the eastern basin^2,16,17^. Although there are no conclusive evidences to support the separation between these purported populations, photo-ID and satellite tracking studies suggest that mixing between them is very low and that the Icelandic feeding grounds are used by the eastern subpopulation^18–20^.

Despite more than 4 decades of complete protection in the North Atlantic, the species has only timidly recovered and its current population appears to be still fragile and is consequently catalogued as Endangered^5^. In this context, accurate information on the historical catches, the status of the subpopulations and their trends, is of prime relevance. Here we analyse the information on blue whales from whaling operations and scientific surveys conducted in the South European Atlantic Shelf ecoregion (sensu^21^; from here called SEAS) and immediately adjacent waters to gain an insight into their demographic trends. To do so, we extracted catch data from the internal landing records of the whaling factories, contrasted them for accuracy with catcher boat logbooks and gunner’s notebooks, and compared the resulting data with those contained in the officially compiled IWC catch data set. Also, for the period 1980–1987 we compile the sightings data from the available logbooks of the catcher boats and from scientific sighting cruises that were carried out during the period 1979–1987. Combining these different sources of information, we attempt to assess the relative abundance of blue whales in the last phases of whaling in the area, the potential impact that the unregulated catches had on the population, and their past and current abundance trends.

## Material and methods

The area covered by this compilation, the SEAS, is defined as the marine ecoregion located off the European continent between the South of the British Isles and the North of Africa^21^. The geographical limits of the area covered were: 48° 30′ N (Normandy)–30° 00′ N (southern limit of the Cadis Gulf) and 18° 30′ W (limit of the area covered by the surveys)–5° 30′ E (Gibraltar Straits). The Mediterranean Sea is not included because the species does not occur in this water mass^22^. We analysed several independent data sources:

1. Standard-type logbooks from the IBSA catcher boats and gunners’ note books: until 1979, the logbooks used by the IBSA whaling fleet had the standard format used in fishing vessels but included with varying degrees of detail the whale catch information in the section of “daily events” (*Acaecimientos*). Recording was in a format-free style text where whales were in most cases only recorded if caught and were identified as either sperm whales or as baleen whales, in the latter case with no distinction between species. The gunners’ notebooks had been kept just for personal recording of catches and bonuses obtained, and were simple listings of catches with dates and species. These two sources of information were of utility for checking the accuracy of the catch data, but could not be used to investigate schooling behaviour or the relative abundance of blue whales.
2. IWC-type logbooks from the IBSA catcher boats: from 1979, the IBSA whaling fleet started to complete a template designed by the IWC, with specific boxes for recording sightings and catches by species as well as for the various operations in which the boat was involved. Sightings recorded were made by whalers with experience in species identification, so they are considered accurate and reliable. These logbooks were used to check the accuracy of landing reports and to calculate the percentage of whales that were lost by catcher boats after being killed. In combination with the sightings surveys data (see below) they were also used to examine school size and composition, as well as to investigate abundance relative to fin whales, the most abundant large whale in the region and the main target of the fishery.
3. Sightings data recorded during line transect surveys conducted by the University of Barcelona during 1981–1987 (“UB-surveys”): these cruises were carried out with the objective of obtaining population estimates of fin whales in the area. Details of sightings of all cetacean species were recorded by a crew composed of both whalers and whale biologists, all with experience in species identification, so they are considered to be accurate and reliable. Cruise details can be found in Aguilar et al.^23^, Sanpera et al.^24,25^, Sanpera and Jover ^26^, and Lens et al.^27^ In combination with the IWC-type logbook data (see above) they were used to examine school size and composition, as well as to investigate abundance relative to fin whales.
4. Blue and fin whale catch data: catch data from the whaling companies operating between 1921 and 1985 in the region (Spain, continental Portugal and Spanish Morocco) were taken from a previous review of local data sources conducted by Aguilar^14^ and checked against the IWC catch dataset^4^. Those from the three whaling stations that operated in NW Spain during the 1951–1986 period, Caneliñas (1951–1986), Balea (1985) and Morás (1965–1975), were extracted from the internal landing records of the managing company, Industria Ballenera SA (IBSA), and checked for accuracy against the standard format and IWC-type logbooks available from the IBSA catcher boats, and then used to correct Aguilar^14^ and Allison^4^ databases. Data from the unregulated operations of the *Sierra* and the *Tonna* were taken from published sources^28^. The distribution of the body lengths collected by the IBSA whaling company during 1974–1985 were compared to those from the database of biological measurements collected by scientists from the University of Barcelona during the same period (UB database) to assess potential biases in IBSA measurements. Data normality of body length distributions was assessed through the Shapiro–Wilk test, and differences between distributions and medians were tested using the U Mann Whitney test and the Mood's median test for independent samples, respectively. Additionally, body length distributions for the different operations and periods were also investigated visually to identify oversized individuals that could indicate inaccuracies in measurements or in the species assignment in databases.
5. Internal correspondence from two Norwegian whaling companies that operated in the region: the *Compañía Ballenera Española* (Lorentz Bruun and von der Lippe, Tønsberg), which conducted whaling during 1921–1927 both in the Straits of Gibraltar and off NW Spain, and *AS Hektor* (N. Bugge,Tønsberg)/Industrial Marítima S.A., which operated during 1934–1954 in the Gibraltar Straits. This documentation was examined at the Vestfold Museene (Sandefjord, Norway).

The loss rates of whales that were killed, that is, the proportion of whales that were actually harpooned and secured either to the boat or to a buoy, but were subsequently lost due to bad weather, sinking of corpse, parting of the securing chains, or other reasons, was calculated as a percentage of those that were landed at the whaling stations. The paucity (n = 15) of loss events precluded stratification by period, comparison between catcher boats or companies, or other more sophisticated analyses. Because neither the logbooks nor the gunners’ notebooks reported the loss of whales that had been struck but escaped, or those which were lost before being secured to the boat or to a buoy, we equated the number of whales that were successfully secured to those that were killed. The resulting numbers likely underestimate actual kills but, because the efficiency of harpooning techniques was high, we assume that any difference existing between the number of struck whales, and thus possibly killed, and that of secured whales would be small.

Because no direct abundance estimates for blue whales are available for the region, we produced a rough indication of abundance to assess the potential impact of catches on the population as well as its demographic time-trends. To do so, we compared the number of catches and sightings of blue whales obtained from the catcher boat logbooks and scientific surveys with those of fin whales, a species whose abundance is relatively well known thanks to specifically-designed line-transect boat surveys conducted in the region (see below in the “Discussion” section). In doing so, we made the following assumptions: (1) that the two species were equally attractive to whalers; (2) that the two species presented similar difficulty of capture; and (3) that the two species had a similar sightabilty rate. Until 1980, first year when blue whale protection effectively came into force in Spain, the first assumption seems valid and substantiated by the personal experience of AA, who conducted abundant fieldwork during 1977–1985 onboard the catcher boats. Being the products of both species commercially indistinguishable, the only potential bias would have been caused by the larger maximum body size of adult northern hemisphere blue whales (26–27 m, total body length) as compared to that of northern hemisphere fin whales (22–23 m, total body length)^29^. However, because only juveniles of the species appear to occur in the SEAS (see below) and their body size approaches that of adult fin whales, the effect of such bias is probably reduced. If indeed existing, the bias would tend to slightly increase the catchability rate of blue whales and, with this, the relative abundance of this species in relation to that of fin whales, thus pushing upwards its abundance estimates. Whatever the case, it should be noted that the potential effect of this bias would only affect the estimates obtained from the catch data, but not those derived from the sighting rates of the catcher boats or from the scientific surveys because the efficiency at which the two species are sighted is indistinguishable. Thus, their sightability by observers, that is, the combination of the perception bias (proportion of whales that may be missed by observers) and the availability bias (proportion of diving whales that are not visible) are very similar for blue and fin whales^30^, and this gives grounds to rely on the representativeness of the sightings ratio between the two species. Considering this, a blue whale-fin whale catch ratio was estimated as the number of blue whales for every 100 fin whales caught for the different periods and areas in the SEAS ecoregion.

## Results

Table S1 presents a summary of the information contained in the logbooks and gunners’ notebooks from the earlier period (standard-type). We examined 13 logbooks of this type from 10 whaling seasons that took place between 1953 and 1980. The logbooks did not always record the complete whaling season; in some cases, this was so because the boat only worked part of the season, while in other cases the logbook was incomplete. Table 1 presents the details of the IWC-type logbooks and the line transect survey forms examined, with a summary of the sightings of fin and blue whales therein recorded. The IWC-type logbooks (n = 11) covered 6 complete whaling seasons that took place between 1979 and 1985 and which, although varying between years, usually lasted from early May to late November. The median Julian date when blue whales were sighted was 248 (4/5 September), with 25–75 percentiles = 223–278 (11/12 August–5/6 October). The line transect survey forms corresponded to 6 cruises carried out between 1979 and 1987; all were conducted from mid-July to mid-September to match the period of maximum fin whale abundance in the area^31^. Table S2 shows the details of the blue whale sightings recorded in both the IWC-type logbooks and the line transect survey forms. In the blue whale sightings from which information on school composition was available (n = 71), most sightings corresponded to single blue whale individuals (90.5% of cases; mean school size = 1.1; SD =  ± 0.34; maximum = 3). However, in 38% of the sightings (n = 27) the blue whales, either single or in pairs, were seen forming a mixed school with one or more fin whales.

**Table 1:** Details of the IWC-type logbooks and the line transect surveys, with results of sightings, school size, and relative sighting rates (%) for fin and blue whales.

Vessel	Year	Start date	Final date	Total effort days	Number of fin whale schools sighted	Number of fin whales sighted	Mean fin whale school size	Number of blue whale schools sighted	Number of blue whales sighted	Mean blue whale school size	Blue/fin whale sightings ratio (%)	Blue/fin whale numbers ratio (%)
**Sightings from catcher boat logbooks**												
IBSA UNO	1979	23 May	15 Aug	84	130	286	2.20	2	5	2.50	1.54	1.75
IBSA TRES	1981	7 June	28 Nov	175	126	696	5.52	1	1	1.00	0.79	0.14
Lobeiro	1981	20 June	29 Nov	163	99	366	3.70	4	4	1.00	4.04	1.09
IBSA UNO	1982	22 May	28 Sept	129	78	318	4.08	1	1	1.00	1.28	0.31
IBSA TRES	1982	1 May	27 Sept	150	129	445	3.45	9	11	1.22	6.98	2.47
IBSA TRES	1983	1 June	29 Oct	151	191	445	2.33	14	15	1.07	7.33	3.37
IBSA UNO	1983	1 June	28 Oct	150	103	319	3.10	12	12	1.00	11.65	3.76
IBSA UNO	1984	24 June	29 Oct	127	98	397	4.05	9	9	1.00	9.18	2.27
IBSA TRES	1984	24 June	29 Oct	127	124	287	2.31	16	17	1.06	12.90	5.92
IBSA UNO	1985	16 Aug	21 Oct	66	77	158	2.05	2	2	1.00	2.60	1.27
IBSA TRES	1985	24 July	19 Oct	87	110	300	2.73	1	1	1.00	0.91	0.33
Total				1409	1265	4017	3.18	71	78	1.10	5.61	**1.94**
**Sightings from line transect surveys**												
BALLENA 1	1981	2 September	19 September	18	23	38	1.65	2	2	1.00	8.70	5.26
BALLENA 2	1982	1 August	30 August	29	37	52	1.41	3	5	1.67	8.11	9.62
BALLENA 3	1983	1 August	30 August	30	40	89	2.23	1	1	1.00	2.50	1.12
BALLENA 4	1984	15 July	14 August	30	34	59	1.74	1	2	2.00	2.94	3.39
BALLENA 5	1985	1 August	13 August	13	5	9	1.80	1	1	1.00	20.00	11.11
NASS-87	1987	6 July	3 August	20	101	137	1.36	0	0		0.00	0.00
Total				140	240	384	1.60	8	11	1.38	3.33	**2.87**

**Figure 2:**
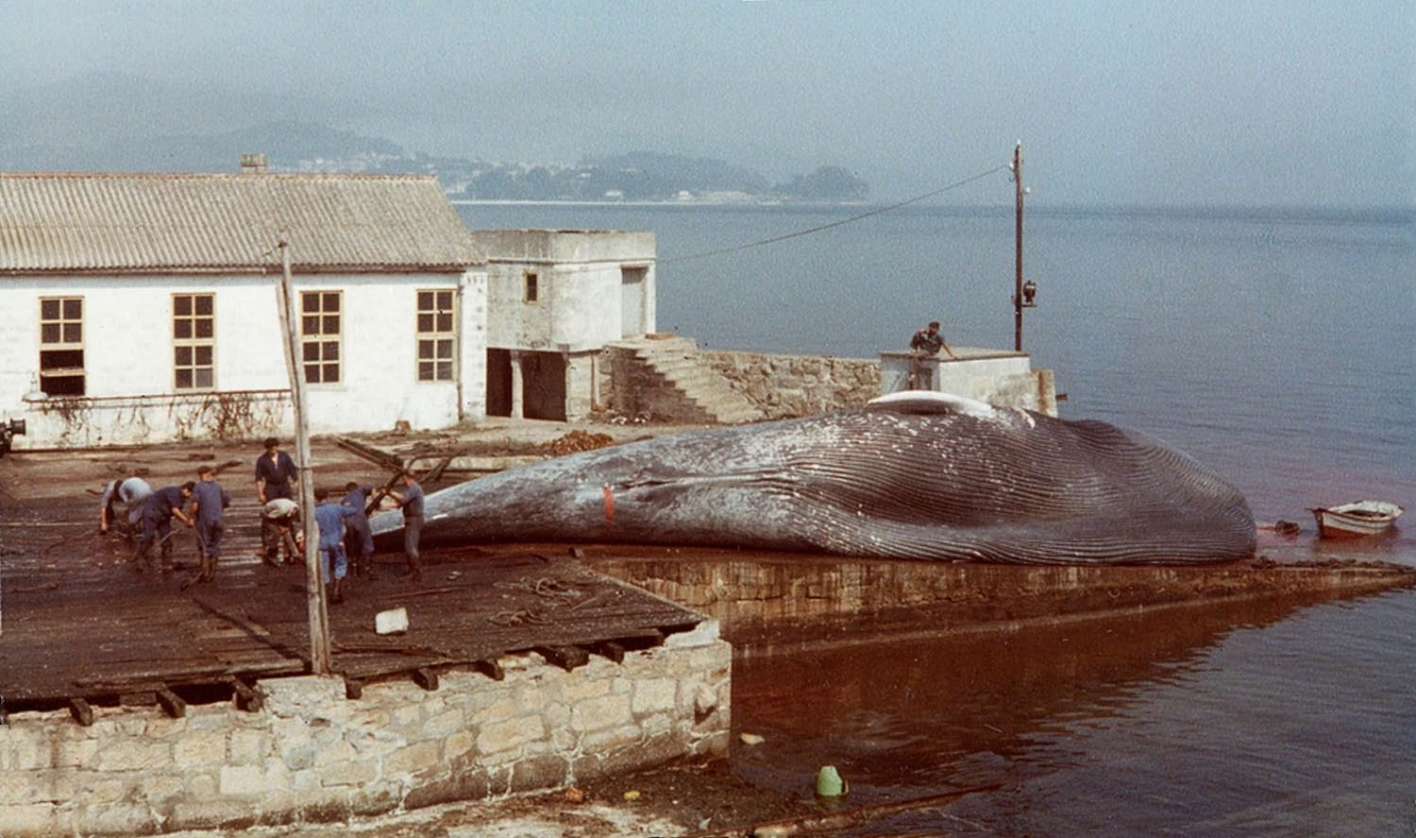
The 22,40 m long blue whale caught by the catcher boat *IBSA DOS* on 24 July, 1978, and brought to the Cangas (Balea) land factory for flensing.

Table 2 provides the details of the blue whale catches recorded in the various whaling operations that took place in the waters around the Iberian Peninsula during the twentieth century. In total, the catches number 60 individuals over a period of 55 years, that is, 1.01 individuals/year. About half (31) of these catches were previously unknown and are not contained in the IWC catch dataset. In many of these cases (24) the whales were indeed recorded as caught but incorrectly identified as fin whales, but 7 were not recorded at all. With the exclusion of one individual of unknown sex measuring 9.10 m, the mean body length ± standard deviation of the blue whales caught were 20.44 m ± 1.87 m (20.15 m ± 1.95 m for males and 20.55 m ± 1.79 m for females). Photographs from two blue whales caught could be obtained; in both cases the species identification was correct (see Fig. 2 for one of them).

**Table 2:** Details of the blue whale catches recorded, with catch date, body length, sex, and presence/absence (Y/N) of foetus, company responsible for the capture, factory in which the individual was flensed, source, and whether and how the catch is reported in the official IWC statistics database^4^.

Year	Month	Day	Julian date	Body length (m)	Sex	Foetus	Company	Factory	Source	Reported in IWC statistics
**Gibraltar straits**										
1924	?	?		?	?	?	Corona S.A	Rey Alfonso floating factory	IWC statistics	Not reported
1948	1	3	3	22.00	♂	N	Hektor A/S	Benzú	IWC statistics	Yes
1949	?	?		?	?	?	Hektor A/S	Benzú	INE^57^	Not reported
1954	4	14	103	20.90	♀	N	Industrial Marítima SA	Benzú	IWC statistics	Yes
1954	4	21	110	19.00	♂	N	Industrial Marítima SA	Benzú	IWC statistics	Yes
1954	4	22	111	19.00	♀	N	Industrial Marítima SA	Benzú	IWC statistics	Yes
1954	4	29	118	20.50	♀	N	Industrial Marítima SA	Benzú	IWC statistics	Yes
1954	4	30	120	19.50	♂	N	Industrial Marítima SA	Benzú	IWC statistics	Yes
**Central Portugal**										
1925	?	?		?	?	?	Soc. Portuguesa de Pesca de Cetáceos	Professor Gruvel Floating factory	IWC statistics	Yes
1951	5	27	147	18.00	♀	N	Soc. Portuguesa de Pesca de Cetáceos	Setúbal	IWC statistics	Yes
**NW Spain**										
1925	?	?		?	?	?	Compañía Ballenera Española	Caneliñas	IWC statistics	Yes
1925	?	?		?	?	?	Compañía Ballenera Española	Caneliñas	IWC statistics	Yes
1926	6	11	162	?	?	?	Compañía Ballenera Española	Caneliñas	CBE correspondence	Not reported
1958	4	25	114	19.90	♀	N	Industria Ballenera SA	Caneliñas	IBSA factory logs	Yes
1958	7	5	186	17.50	♂	N	Industria Ballenera SA	Caneliñas	IBSA factory logs	Yes
1959	10	19	292	9.10	?	N	Industria Ballenera SA	Cangas	IBSA factory logs	Yes
1962	9	31	274	20.70	?	N	Industria Ballenera SA	Caneliñas	IBSA factory logs	Not reported
1964	4	11	100	23.30	?	N	Industria Ballenera SA	Caneliñas	IBSA factory logs	Yes
1964	7	19	200	22.40	♂	N	Industria Ballenera SA	Caneliñas	IBSA factory logs	Yes
1965	8	16	228	20.80	♂	N	Industria Ballenera SA	Cangas	IBSA factory logs	Reported as fin whale
1966	7	1	182	21.00	♀	N	Industria Ballenera SA	Morás	IBSA factory logs	Reported as fin whale
1967	5	21	141	24.00	♀	N	Industria Ballenera SA	Caneliñas	IBSA factory logs	Yes
1968	9	14	257	20.80	♂	N	Industria Ballenera SA	Morás	IBSA factory logs	Reported as fin whale
1968	9	27	270	20.40	♀	N	Industria Ballenera SA	Caneliñas	IBSA factory logs	Reported as fin whale
1969	9	17	260	23.00	♂	N	Industria Ballenera SA	Caneliñas	IBSA factory logs	Yes
1970	8	24	236	23.40	♀	N	Industria Ballenera SA	Caneliñas	IBSA factory logs	Yes
1971	7	30	211	23.20	♀	N	Industria Ballenera SA	Caneliñas	IBSA factory logs	Yes
1971	9	13	256	18.50	♂	N	Industria Ballenera SA	Cangas	IBSA factory logs	Reported as fin whale
1971	10	10	283	17.70	♂	N	Industria Ballenera SA	Cangas	Gunner’s records	Not reported
1972	11	17	321	18.00	♂	N	Industria Ballenera SA	Caneliñas	IWC statistics	Reported as fin whale
1973	6	22	173	23.50	♀	N	Industria Ballenera SA	Morás	IBSA factory logs	Yes
1974	8	12	224	19.70	♀	N	Industria Ballenera SA	Caneliñas	IBSA factory logs	Reported as fin whale
1974	10	9	282	19.70	♀	N	Industria Ballenera SA	Cangas	IBSA factory logs	Reported as fin whale
1974	10	10	283	21.00	♀	N	Industria Ballenera SA	Caneliñas	IBSA factory logs	Reported as fin whale
1974	10	12	285	19.30	♂	N	Industria Ballenera SA	Caneliñas	IBSA factory logs	Reported as fin whale
1975	7	25	206	18.80	♂	N	Industria Ballenera SA	Cangas	IBSA factory logs	Reported as fin whale
1975	9	18	261	19.00	♀	N	Industria Ballenera SA	Caneliñas	IBSA factory logs	Reported as fin whale
1975	9	23	266	18.60	♀	N	Industria Ballenera SA	Caneliñas	IBSA factory logs	Reported as fin whale
1975	10	8	281	18.60	♀	N	Industria Ballenera SA	Caneliñas	IBSA factory logs	Reported as fin whale
1975	10	9	282	21.90	♀	N	Industria Ballenera SA	Caneliñas	IBSA factory logs	Not reported
1975	10	26	300	18.40	♂	N	Industria Ballenera SA	Caneliñas	IBSA factory logs	Reported as fin whale
1975	10	28	302	19.40	♂	N	Industria Ballenera SA	Caneliñas	IBSA factory logs	Reported as fin whale
1976	8	9	221	19.20	♀	N	Industria Ballenera SA	Cangas	IBSA factory logs	Yes
1976	9	23	266	19.30	♀	N	Industria Ballenera SA	Cangas	IBSA factory logs	Reported as fin whale
1977	8	11	223	22.10	♂	N	Industria Ballenera SA	Cangas	IBSA factory logs	Yes
1977	9	10	253	23.70	♂	N	Industria Ballenera SA	Cangas	IBSA factory logs	Yes
1977	9	14	257	20.30	♂	N	Industria Ballenera SA	Cangas	IBSA factory logs	Reported as fin whale
1977	9	18	261	18.10	♂	N	Industria Ballenera SA	Cangas	IBSA factory logs	Reported as fin whale
1977	9	20	263	18.90	♂	N	Industria Ballenera SA	Cangas	IBSA factory logs	Reported as fin whale
1977	10	31	305	20.50	♀	N	Industria Ballenera SA	Cangas	IBSA factory logs	Reported as fin whale
1978	7	8	189	22.80	♂	N	Industria Ballenera SA	Caneliñas	IBSA factory logs	Yes
1978	7	24	205	22.40	♂	N	Industria Ballenera SA	Cangas	IBSA factory logs	Yes
1978	7	28	209	24.10	♀	N	Industria Ballenera SA	Caneliñas	IBSA factory logs	Yes
1978	10	15	288	19.10	♀	N	Industria Ballenera SA	Caneliñas	IWC statistics	Reported as fin whale
1978	11	21	325	19.00	♀	N	Industria Ballenera SA	Caneliñas	IWC statistics	Reported as fin whale
1978	11	25	329	21.10	♀	N	Industria Ballenera SA	Caneliñas	IWC statistics	Reported as fin whale
1978	?	?		?	?	?	M. V. Sierra	Floating factory	Best (1992)^54^	Yes
1978	?	?		?	?	?	M. V. Sierra	Floating factory	Best (1992)^54^	Yes
1978	?	?		?	?	?	M. V. Sierra	Floating factory	Best (1992)^54^	Yes
1979	7	29	210	19.60	♀	N	Industria Ballenera SA	Caneliñas	IBSA factory logs	Not reported

Figure S1 shows the body length distributions of fin whales caught during 1974–1985 as measured by the IBSA company and as measured by the UB scientists. In both cases body lengths of both males and females were not normally distributed. Males did not show significant differences (*p* > 0.5) between the two groups of samples neither in median (18 m) nor in distribution, but females from the official IBSA database had a significantly (*p* < 0.05) higher median body length (19.1 m) than those from the UB database (18.9 m).

Figure S2 shows the body length distributions of fin whales caught by the various periods and companies that operated around the Iberian Peninsula. Because for of most operations sample size was too scant to conduct reliable statistical analysis, no attempt was made to test for differences in distribution or median values. However, the right tail of the length distributions was examined to identify potentially oversized individuals suggestive of catches of blue whales. The data from Getares for 1950–1959, Benzú for 1947–1954, and Setúbal for 1950–1951 showed individuals larger than 23 m, with distribution tails that departed from an expectable distribution of fin whale body lengths, i.e. such as that followed by the IBSA official or the UB biological measurements datasets.

The statistics available for the 1920s only record a few catches of blue whales, which probably reflects the scarcity of the species, although it is also likely that a few blue whales may have been caught and reported as fin whales (see “Discussion” section). Afterwards, the catch statistics compiled here register the capture of blue whales apparently reliably. In the case of the period 1953–1985 for NW Spain, the catch and sightings information contained in the catcher boat logbooks and in the gunner’s notebooks matched the landing records from the managing company, Industria Ballenera SA (IBSA) with only minor discrepancies, mostly in the date of capture, and are thus considered accurate. A fin whale-hybrid caught in 1984^32^ was recorded as a fin whale, with no indication of its blue-fin whale mixed origin.

The landing records did not register the whales caught but lost after capture. The loss rates were calculated from the logbooks that contained specific details on losses (*Temerario*, 1953; *Lobeiro*, 1960; *Carrumeiro*, 1978 and 1980; *Lobeiro*, 1980; *IBSA TRES*, 1982, 1983 and 1985), and the rates calculated against the sperm and baleen whale catches recorded for the boats and periods involved. The resulting loss rate estimate was 3.2 for sperm whales (4 out of 126 catches) and 2.3% for baleen whales (15 out of 654 catches). Although none of the loss cases was identified as involving a blue whale, the rates from other baleen whales (mostly fin whales) can be reasonably applied also to blue whales. After correcting for losses, the resulting total number of whales actually killed was estimated at 61.4 blue whales along a period of 55 years, that is, 1.12 individuals/year.

Table 3 details the blue whale-fin whale catch ratio for different periods and areas in the SEAS ecoregion. The data for the nineteenth century were extracted from a study on the whaling operations conducted by open-boat whalers in the southern fringe of the SEAS^33^. The ratio for the 1930s has not been calculated because only 69 fin whales and no blue whales were caught during this decade, and these numbers were considered insufficient to produce a representative result.

**Table 3:** Blue whale-fin whale catch and sightings ratios for different periods and areas in the SEAS ecoregion.

Period	Source	Straits of Gibraltar	Central Portugal	NW Spain
Nineteenth century	Sightings from open-boat whalers	5.90		
1920s	Catch statistics	0.02	0.15	0.18
1930s	Catch statistics			
1940s	Catch statistics	1.48		
1950s	Catch statistics	1.60	2.33^a^	0.47
1960s	Catch statistics			0.91
1970s	Catch statistics			1.34
1980s	Sightings in IWC-type logbooks			1.94
	Sightings in UB-surveys			2.87

The ratio is calculated as the number of blue whales caught or sighted per 100 fin whales caught or sighted (%). Data from the nineteenth century are collected from the literature^30^.

^a^In the 1950s, whaling in central Portugal (Setúbal) was little active and statistics only include the catch of 43 fin whales and 1 blue whale^23^, so the ratio for this particular decade is considered little representative.

## Discussion

### Exploitation

Despite there perhaps having been some exceptional catches of blue whales by open-boat whalers, such as the one made by the American whaling bark *Benjamin Franklin* in 1864^33^, the species was not the focus of exploitation in the region until the onset of modern whaling in the 1920s. For the early period (1920–30s) there are not detailed whaling statistics but only aggregated numbers of catches per company and factory. These records include one blue whale taken by the *Rey Alfonso* floating factory in the Gulf of Cadiz in 1924, one by the Professor Gruvel (*A/S Congo*) floating factory off Setúbal in 1925, and two by the Compañía Ballenera Española off NW Spain and brought to the Caneliñas land factory in 1925^14^. However, when listing the bonuses that this last company payed to the crew, on June 11 1926 Carl Herlofson, the manager of the company in Spain, stated to the Norwegian direction in Tønsberg that gunner Aksel Johansen had to receive a bonus for the capture of a blue whale^34^. This blue whale capture was not included in the statistics, or was mislabelled as a fin whale and, although this was the only case of this nature that we could identify, it may suggest that a few additional blue whales were also caught and not reported. However, the above mentioned case is the only mention of a blue whale catch in the very abundant available correspondence and documentation from these operations, which consistently reported catches of only sperm, fin and sei whales (though the latter was sometimes confused with Bryde’s whales). Apparently confirming this, the rich photographic material that has survived from these operations^14^ never shows a blue whale among the catch. Given that in the 1920s and 1930s the blue whale was not protected but, on the contrary, was a much sought-after species, there is no reason why its catch would have been hidden, particularly in the internal documents and correspondence of the company. Therefore, the actual occurrence of such unreported catches, if they exist at all, is considered to be very small. Conversely, the comparison of the official datasets with the internal records of the Spanish whaling companies that operated during 1944–1985 permitted to identify 31 blue whales that had been not reported, thus doubling the official catch numbers (n = 29). In many cases, the catch of these whales was declared, but they were incorrectly reported as fin whales. It is unclear why in some cases the companies correctly reported the blue whale catches while in others they produced an incorrect identification. Until 1979, when most of these unreported catches occurred, Spain was not part of the IWC so it was not bound by any catch limit or whaling regulation; indeed, some misled records are included in yearly reports that do include correctly declared blue whale catches (e.g. in years 1971, 1975, 1976, 1977 and 1978).

Loss of killed whales appeared to be highly variable between operations. Unskilled crews, the employment of defective equipment or the use of vessels that were not proper catcher boats undoubtedly increased the probability of losing a whale after its harpooning. Even within a given operation, the success in recovering a harpooned whale probably increased with time, as long as crews gained experience and equipment was improved. For example, when the company Marcelino dos Reis Ltda initiated operations in Portugal in 1945, it employed three trawlers equipped with a harpoon gun^12,13^. Owing to the poor quality of lines, the company reported the loss of 32 out of 79 harpooned and secured whales in the first year of operation, that is, 40% of catches. The rate barely improved in the following season, with 22 whales lost out of 152, that is, 14,5% of catches^35^. However, loss rates of such magnitude were certainly not the rule, and at least in the more modern operations they were generally low. Thus, the logbooks here examined for the period 1953–1985 indicate a loss rate for baleen whales of 2.3%, a value that, although it is mostly derived from the fin whale catch, should also apply to the blue whale because of its similar body mass, shape and buoyancy. Given that the statistics only reflect the whales that were landed, the blue whale catch numbers here reported should be multiplied by a correction factor of 1.023 to estimate the actual number of whales killed, thus resulting in a take of 61.38 blue whales along a period of 55 years, that is, approximately one individual/year.

The comparison between the IBSA body length distributions of the fin whales caught during 1974–1985 and those reported of the UB database, distributions and median body length are similar for males, but for females the median was 20 cm longer in the first database than in the second. This small difference can be explained by the fact that, while IBSA workers measured the whale’s body length from tip of the snout to the tip of tail flukes, UB scientists did that from the tip of the snout to the notch of flukes (AA personal observation). The fin whale body length data from the other operations, although often limited in sample size, showed distributions and medians that reasonably matched the IBSA much larger and verified dataset. However, the fin whale body length distributions from Getares (1950–1959), Benzú (1947–1954), and Setúbal (1950–1951) contained a number of individuals longer than 23 m, that is*,* excessively large to be fin whales. This may be taken as an indication that these individuals were indeed misreported blue whales. However, the shape of the right tails of these distributions depart from what would be an expectable distribution of body lengths, that would end with a progressive decline in extreme values, so they are considered to be unreliable measurements. For example, for Getares (1950–1959) 4 out of 8 whales taken would have purportedly been blue whales (body length larger than 23 m), a proportion unreasonable for the fin whale/blue whale abundance ratio in the area. Unfortunately, clarification and retrospective correction of databases is unfeasible with the current level of information. However, the existence of these oversized whales must be borne in mind and it cannot be ruled out that the number of blue whales actually taken from the population was indeed somewhat higher than the above estimate.

The mean body length of individuals reported as blue whales was somewhat lower for both sexes than corresponding values given by Risting^36^ for higher latitudes of the North Atlantic (Greenland, West coast of Ireland, Shetland and Newfoundland). The difference may be due to a lower selectivity of whalers when catching whales in the SEAS but the statistics do not contain any specimen larger than 24 m, which in other North Atlantic regions were frequent, despite the fact that whalers would certainly have tried by all means to capture a specimen of this size. The apparent lack of large individuals may be due to a certain population stratification with latitude, with juveniles tending not to penetrate such high latitudes as adults, as it has been observed in the South Atlantic, where a gradient in sizes and a greater presence of juveniles in southern latitudes apparently occurs^36,37^. Moreover, the statistics regularly recorded the presence and length of foetuses^4^, but none of the recorded blue whales caught was apparently pregnant, again supporting the absence of fully adult individuals. One caught whale, however, was only 9.1 m long, which indicates that this was a calf.

### Status of the population

The blue whale does not appear to have ever been abundant in the temperate waters of the eastern North Atlantic. Strandings of blue whales along the shorelines of northwestern Africa, western Spain, continental Portugal and southwestern France are unusual in both historical and more recent times^22,38–40^. Consistent with this low abundance, the catches of this species in the SEAS only represented a very small fraction of the total catches of the species conducted in the North Atlantic^4^. Because a line-transect model requires a minimum number of sightings to be fitted, a consequence of such low abundance of blue whales is that the various line-transect surveys carried out in the region that have served to produce population numbers for other cetacean species, mostly the fin whale^23–27,41–44^, have been unable to collect enough data for blue whales to produce an approximate estimate of abundance or even density, as it would have been ideal. However, an insight into the relative abundance of this species can be obtained through the ratio between the catches or sightings of blue whales as related to those of fin whales. It should be noted that the resulting numbers of such exercise cannot be considered population estimates for blue whales, but just rough approximations to the relative abundance of the species.

Previous to the onset of blue whale exploitation by modern whalers, the American open boat vessels operating during 1862–1889 in the Gibraltar Straits ground had a sighting ratio of 5,9 blue whales for every 100 fin whales, as shown by the data recorded in the whaling vessels’ logbooks^33^. In the twentieth century there is no information available on sighting rates until the 1980s, but in the 1920s the catch ratio of blue whales for every 100 fin whales was only 0.02–0.18% depending on the area, that is, an order of magnitude lower than the already low pre-exploitation sighting rates observed in the open-boat vessels’ logbooks. This undoubtedly reflects the severe effects of the massive blue whale catches that occurred in the North Atlantic during the last decades of the nineteenth century and the early decades of the twentieth century^6^. From the 1940s and up to the 1970s, the catch ratio between the two species increased to 0.47–1.6%, probably as a consequence of the opposing trends in their abundance: while the blue whale benefited from the ban in its exploitation approved by the IWC^8^, the fin whale replaced the blue whale as a main target and whaling pressure on that species suddenly increased^1^. It is interesting to note that the ratio was not uniform between areas, and during the second half of the twentieth century it was 3–4 times higher in the southern whaling grounds than off NW Spain. It is very likely that this does not reflect a higher abundance of blue whales in the southern grounds but rather a much reduced density of fin whales there as a consequence of the collapse of the Gibraltar Straits subpopulation due to the unsustainable level of catches that had been made in the 1920s^14,45^.

In NW Spain, where the fin whale population was relatively abundant despite 6 decades of almost continuous exploitation^14^, the blue whale/fin whale ratio showed a progressive increase with time. In 1979 Spain joined IWC and stopped taking blue whales. No catch ratios can thus be calculated, but the sightings ratios from both the IWC-type logbooks and the UB-surveys shows a continued rise in the proportion of blue whales sighted as related to that of fin whales. Although the rate appears somewhat higher in the UB-surveys (2.86%) than in the sightings performed by whalers (1.94%), this apparent difference should be taken with caution because sample size was much lower in the first database than in the second (n = 248 vs n = 1336) and thus would be expected to be less accurate.

A line transect survey conducted in 1989 resulted in a fin whale abundance estimate of 17,335 individuals (95% CI 10,400–28,900), a figure that is considered robust^46^ because the cruise covered the whaling grounds and adjacent waters, and the sighting rates obtained were consistent with those found in surveys from the 1980–1990s^30,41–47^ covering smaller or partially overlapping areas. A comparable survey conducted in 2016 in a somewhat different and larger area^43^ resulted in a fin whale abundance estimate of 18,142 individuals (95% CI 9796–33,599). This latter survey was conducted during 4–28 July, that is, about 2 months earlier than the period when blue whales visit the area, and concentrated its effort in shelf waters with relatively sparse coverage of offshore waters, all which combined to produce insufficient data to assess blue whale abundance and is considered suboptimal for comparative purposes^43^. If the blue whale/fin whale sighting rate obtained in the catcher boat and survey observations of the 1980s is applied to the 1989 Bucklands’s et al.^46^ estimate, the resulting abundance of blue whales in the SEAS ecoregion is 337 individuals (95% CI 202–561) when applying the 1.94% value, and 497 individuals (CI 298–828) when applying the 2.86% value. These figures are much higher than the abundance of only a few tens suggested for the area by Cooke^5^. Taking into account the above abundance numbers, the catches conducted during the period 1921–1979 by the Iberian whaling operations (1.12 individuals/year) are not expected to have had a significant effect on the trajectory of the population.

During summer, blue whale relative abundance in the NE Atlantic tends to increase northwards. Thus, the blue whale/fin whale ratio was found to be 1.94–2.86% off NW Spain, 7.7% in the Irish Porcupine Seabight^48^, and 9.75% off Iceland^49^. Iceland, which appears to be a main feeding ground for the eastern subpopulation^19^, is where the blue whale appears to reach its highest density. In this area, the sightings from both the whaling boats and line transect cruises indicate that the population has been increasing during the last decades at a mean annual rate of 4–5.2%^30,50^, values that are somewhat lower than the 7.3% estimated for Antarctic blue whales^51^. As a result of this raise in abundance, the population off Iceland was estimated in 2015 to be at about 3000 individuals (95% CI 1377–6534), that is, about six times higher than the estimate for 1989^52^. If these annual rates of increase are applied to the gross estimates of abundance in the SEAS estimated for 1989, it can be derived that the number of blue whales in the region must be currently over a thousand individuals. Such an increase would be consistent with the progressive penetration of the species in the shelf waters and the rise in strandings and sightings that has been reported in the last decade in NW Spain^53–55^. However, despite the apparent increase in abundance the school size has remained very low, with most sightings being of mixed schools with fin whales or of single individuals, as it is the case in other depleted blue whale populations^56^.

Because the population estimates conducted in the various regions of the North Atlantic derive from line transect surveys conducted in all cases during the July–September period, it is reasonable to assume that the figures resulting from them correspond to exclusive aggregations of blue whales and thus can be added; if this is correct, and the annual rates of increase observed in Iceland are applied to the above numbers, the current (2021) size of the overall eastern North Atlantic blue whale subpopulation would presumably be in the order of 4000–5000 individuals. Such abundance levels are higher than those commonly proposed for the basin^5^, although they are still much lower than those which the species once enjoyed in the North Atlantic. The total number of blue whales killed in this ocean during the first decades of exploitation was likely in the range 15–20,000^5^. As a consequence, its initial population size was over such threshold and the current levels of abundance would still be below one third of pre-exploitation numbers. Whatever the case, these are proximate calculations that should not be regarded as robust estimates of population size but can be used as a guidance to infer rough levels of abundance of the species until especially dedicated line-transect surveys or photoidentification studies provide more accurate numbers.

## Supplementary Information

Supplementary Information.
