# Supplementary material for: Unreported catches, impact of whaling and current status of blue whales in the South European Atlantic Shelf
Source: Sci Rep. 2022 Mar 31;12:5491. doi: 10.1038/s41598-022-09570-6 (PMC8971400; doi:10.1038/s41598-022-09570-6)
Supplement: Supplementary file 1 — Supplementary Information. [file 41598_2022_9570_MOESM1_ESM.pdf]

SUPPLEMENTARY DATA

Unreported catches, impact of whaling and current status of blue whales in the South European Atlantic Shelf

Alex Aguilar* and Asunción Borrell

Department of Evolutionary Biology, Ecology and Environmental Sciences, Faculty of Biology, Universitat de Barcelona, 08028 Barcelona, Spain

Institut de Recerca de la Biodiversitat (IRBio), Universitat de Barcelona, 08028 Barcelona, Spain

Table S1.- Details of the logbooks of standard format examined.

Vessel	Year	Start date	Final date	Total effort days	Remarks
Temerario	1953	9 May	12 Dec	287	
Lobeiro	1960	4 Sept	9 Dec	96	
Lobeiro	1972	29 May	9 Nov	164	No positions recorded
Lobeiro	1973	28 May	11 Dec	197	
Lobeiro	1974	25 May	17 Dec	206	
Lobeiro	1975	19 May	16 Dec	211	
Lobeiro	1976	20 May	21 Dec	215	
Temerario	1976	19 May	22 Dec	217	
Lobeiro	1977	16 June	19 Dec	187	
Carrumeiro	1978	20 June	1 Dec	164	
Lobeiro	1978	12 June	1 Dec	172	
Carrumeiro	1980	16 May	20 Oct	157	
Lobeiro	1980	18 July	1 Aug	12	Only few days recorded, no positions

Table S2.- Details of the blue whale sightings made by the catcherboats and by the line-transect and tagging cruises.

Year	Month	Day	Julian day	Number	Boat/cruise	Lat N		Lon W		School composition
1982	7	21	202	1	IBSA 3	42	43	11	10	1 blue whale + 2 fin whales
1981	8	11	223	1	IBSA 3	42	0	10	50	1 blue whale + 2 fin whales
1983	10	21	294	1	IBSA 1	42	50	11	35	1 blue whale
1983	7	2	183	1	IBSA 3	42	43	10	13	1 blue whale
1983	7	25	206	1	Ballena 3	44	48	14	38	1 blue whale
1983	8	2	214	1	IBSA 3	42	55	10	22	1 blue whale
1985	8	3	215	1	IBSA 3	44	3	10	54	1 blue whale
1985	8	3	215	1	Ballena 5	43	23	17	30	1 blue whale
1984	8	7	220	1	IBSA 3	44	36	10	48	1 blue whale
1982	8	8	220	1	Ballena 2	45	41	12	35	1 blue whale
1983	8	8	220	1	IBSA 1	43	13	10	36	1 blue whale
1984	8	12	225	1	IBSA 3	42	53	11	51	1 blue whale
1983	8	15	227	1	IBSA 3	43	49	10	46	1 blue whale
1983	8	16	228	1	IBSA 1	43	5	10	48	1 blue whale
1983	8	19	231	1	IBSA 1	43	50	10	35	1 blue whale
1983	8	21	233	1	IBSA 1	42	50	11	0	1 blue whale
1983	8	23	235	1	IBSA 1	42	20	10	5	1 blue whale
1983	8	24	236	1	IBSA 3	42	36	10	31	1 blue whale
1984	8	24	237	1	IBSA 3	44	11	11	14	1 blue whale
1983	8	25	237	1	IBSA 3	44	1	10	16	1 blue whale
1983	8	27	239	1	IBSA 3	42	29	10	22	1 blue whale
1984	8	29	242	1	IBSA 1	43	52	11	20	1 blue whale
1985	9	10	253	1	IBSA 1	43	54	10	13	1 blue whale
1982	9	12	255	1	Tagging cruise	42	43	10	45	1 blue whale
1983	9	12	255	1	IBSA 1	43	0	10	35	1 blue whale
1982	9	14	257	1	IBSA 3	42	58	10	48	1 blue whale
1982	9	14	257	1	Tagging cruise	42	54	10	50	1 blue whale
1981	9	15	258	1	Ballena 1	41	21	11	30	1 blue whale
1984	9	16	260	1	IBSA 1	43	19	10	25	1 blue whale
1984	9	16	260	1	IBSA 3	43	25	10	25	1 blue whale
1984	9	17	261	1	IBSA 1	43	21	10	33	1 blue whale
1983	9	18	261	1	IBSA 1	43	55	10	30	1 blue whale
1984	9	23	267	1	IBSA 3	43	13	10	48	1 blue whale
1984	9	24	268	1	IBSA 1	43	57	11	0	1 blue whale
1983	10	2	275	1	IBSA 1	43	13	10	53	1 blue whale
1983	10	3	276	1	IBSA 3	42	59	10	26	1 blue whale
1983	10	5	278	1	IBSA 1	43	25	10	22	1 blue whale
1984	10	10	284	1	IBSA 3	42	49	11	49	1 blue whale
1984	10	11	285	1	IBSA 3	43	54	10	50	1 blue whale

1984	10	12	286	1	IBSA 3	43	26	10	31	1 blue whale
1984	10	17	291	1	IBSA 1	44	18	10	32	1 blue whale
1984	10	18	292	1	IBSA 3	42	53	10	20	1 blue whale
1983	10	19	292	1	IBSA 1	43	35	10	29	1 blue whale
1983	10	20	293	1	IBSA 3	42	42	11	45	1 blue whale
1981	6	25	176	1	Lobeiro	43	6	10	55	1 blue whale + 1 fin whale
1983	7	12	193	1	IBSA 3	42	57	10	14	1 blue whale + 1 fin whale
1984	8	3	216	1	IBSA 1	43	19	10	38	1 blue whale + 1 fin whale
1982	8	6	218	2	Ballena 2	44	45	11	20	1 blue whale + 1 fin whale
1983	9	4	247	1	IBSA 3	43	10	10	41	1 blue whale + 1 fin whale
1983	9	5	248	1	IBSA 3	43	27	10	43	1 blue whale + 1 fin whale
1981	9	15	258	1	Ballena 1	41	23	11	30	1 blue whale + 1 fin whale
1984	10	11	285	1	IBSA 3	42	53	11	52	1 blue whale + 1 fin whale
1985	10	14	287	1	IBSA 1	44	0	9	39	1 blue whale + 1 fin whale
1984	8	2	215	1	IBSA 1	42	49	10	28	1 blue whale + 2 fin whales
1984	8	13	226	1	IBSA 3	42	44	12	21	1 blue whale + 2 fin whales
1982	8	14	226	1	Ballena 2	45	29	10	9	1 blue whale + 2 fin whales
1984	9	25	269	1	IBSA 3	44	16	10	27	1 blue whale + 2 fin whales
1984	9	26	270	1	IBSA 3	43	54	10	36	1 blue whale + 2 fin whales
1983	10	22	295	1	IBSA 1	42	40	11	15	1 blue whale + 2 fin whales
1984	10	27	301	1	IBSA 1	42	52	11	31	1 blue whale + 2 fin whales
1984	10	27	301	1	IBSA 3	42	41	11	4	1 blue whale + 2 fin whales
1981	9	4	247	1	Lobeiro	42	40	11	0	1 blue whale + 3 fin whales
1983	10	20	293	1	IBSA 3	42	45	11	47	1 blue whale + 3 fin whales
1984	10	26	300	1	IBSA 1	43	55	11	35	1 blue whale + 3 fin whales
1984	10	27	301	1	IBSA 3	42	45	11	0	1 blue whale + 3 fin whales
1984	8	8	220	2	Ballena 4	44	48	14	50	2 blue whales
1983	7	18	199	2	IBSA 3	42	10	10	9	2 blue whales + 1 fin whale
1982	9	6	249	2	IBSA 3	42	40	10	37	2 blue whales + 1 fin whale
1984	10	26	300	2	IBSA 3	42	52	11	0	2 blue whales + 6 fin whales
1979	8	10	222	2	IBSA 1	44	2	9	50	2 blue whales+1 fin whale
1979	8	10	222	3	IBSA 1	44	2	9	50	3 blue whales
1982	5	27	147	2	IBSA 1	43	15	11	10	No information
1982	6	29	180	1	IBSA 3	43	6	11	9	No information
1982	7	1	182	1	IBSA 3	43	7	10	37	No information
1983	7	16	197	1	IBSA 3	42	53	10	2	No information
1977	8	11	223	1	Lobeiro	44	4	10	1	No information
1982	8	30	242	2	IBSA 3	42	37	10	36	No information
1981	8	31	243	1	Lobeiro	42	50	10	46	No information
1982	9	4	247	1	IBSA 3	42	49	10	39	No information
1982	9	7	250	1	IBSA 3	43	0	10	22	No information
1982	9	12	255	1	IBSA 3	42	45	10	37	No information
1981	9	23	266	1	Lobeiro	42	40	10	30	No information

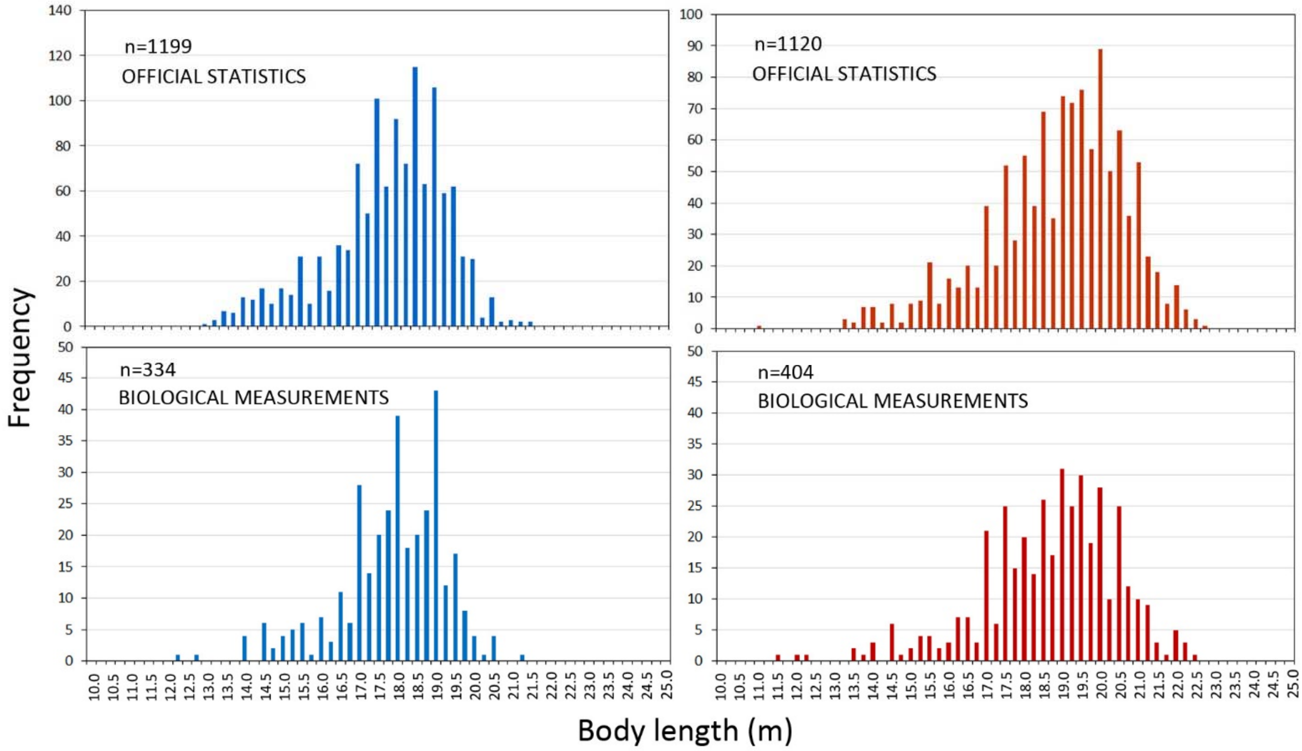

Figure S1: Body length distribution of male (blue) and female (red) fin whales caught during 1974-1985 by the IBSA company. The upper distributions correspond to the data provided officially by the company to the Bureau of International Whaling Statistics and contained in the IWC catch data set. The lower distributions correspond to the data collected by UB biologists and contained in the UB catch data set. The two sources cover the same period but the UB set only include a fraction of the total catch.

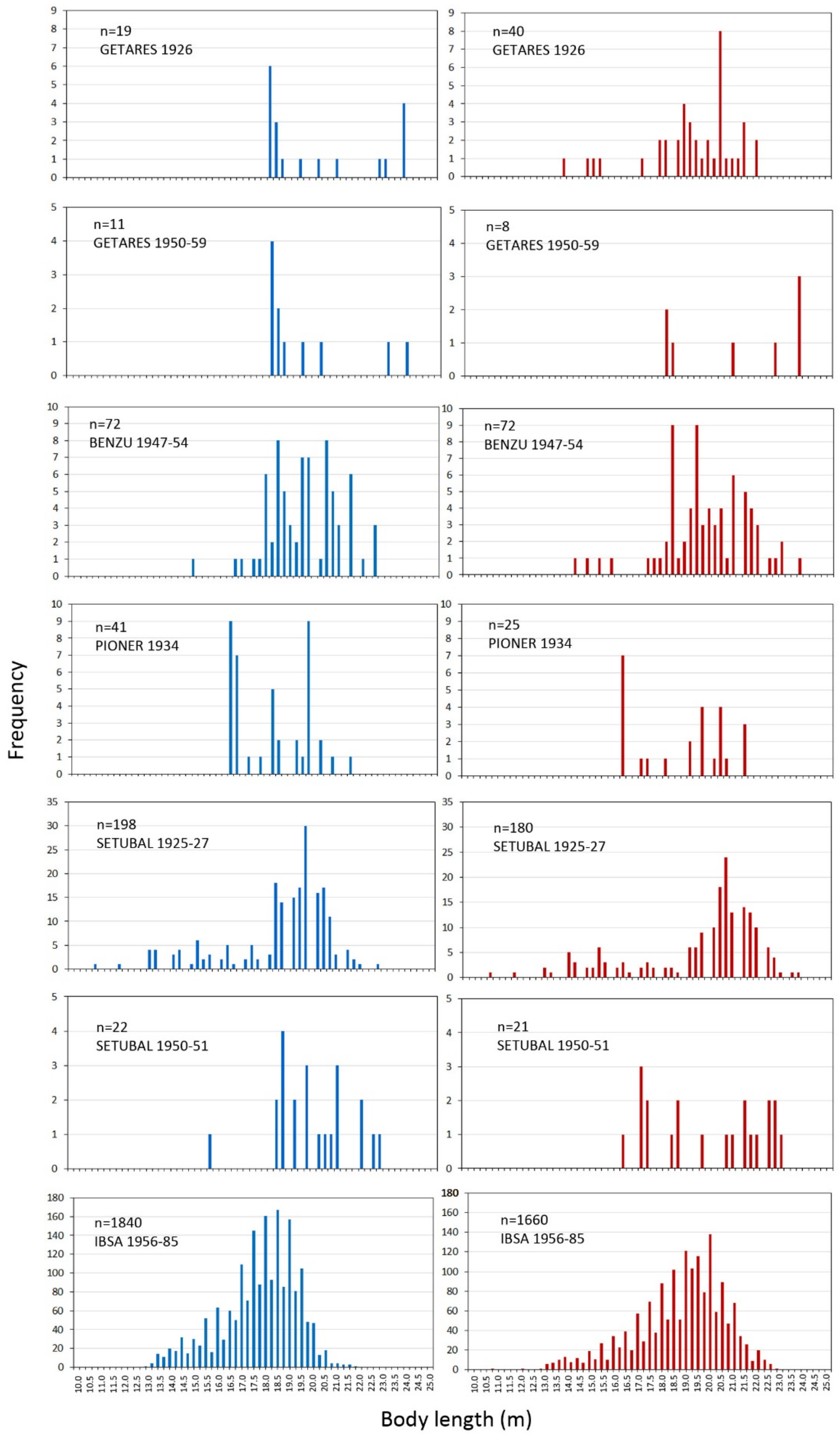

Figure S2: Body length distribution of male (blue) and female (red) fin whales caught in the various periods and whaling companies (for geographical locations see Fig. 1). All data correspond to those provided officially by the companies to the Bureau of International Whaling Statistics and contained in the IWC catch data set.
